# New Drugs and Preparations

**Published:** 1894-06-23

**Authors:** 


### NEW DRUGS AND PREPARATIONS.

[We shall be glad to receive, at our Office, 428, Strand, London, W.C., from the manufacturers, specimens of all new preparations and drugs which may be brought out from time to time.]

### HYPODERMIC USE OF ARSENIC.

Dr. Moyer (Journal American Medical Association, 1893) discusses the advantages of the hypodermic administration of arsenic as compared with giving it by the mouth, and decides emphatically in favour of the former method. Previous observers have generally employed Fowler's solution, but it has the disadvantage of a decided tendency to cause local irritation and abscess. This may be obviated by using arseniate of sodium prepared by heating it to 300 deg. F., to drive off the variable amount of water of crystallization which it contains, and then there is obtained a salt having the fixed composition $Na_2 H As O_4$. A 1 per cent. solution of this salt contains 53 per cent. of the amount of arsenic found in Fowler's solution, so that approximately its dose is twice as great. According to the author arsenic is one of those remedies which is much less toxic when given subcutaneously than when given per os, for the reason that it focuses its action chiefly about the liver, stomach, and upper part of the small intestine; when given by the mouth he says it may make the round of the portal circulation several times before entering the general circulation. Its poisonous effects are due to its action on these organs, and hence are more intense when given by the mouth. Moyer has given more than 500 injections of arseniate of sodium in solutions varying in strength from 1 to 10 per cent., without having ever observed any irritation whatever locally. He usually gives a dose equal to 30 minims of Fowler's solution, and has even gone as high as equal to 75 minims at a single dose; with the latter only the slightest degree of poisonous effects were observed, and none with the former. In his hands he claims that this treatment has given exceptionally good results in chorea; in neurasthenia and paralysis agitans the results were unsatisfactory. He attributes his success in chorea partly to the larger dose given, and partly to the fact that the arsenic comes in contact with the nervous system without having first passed through the liver. Hammond has also largely used arsenic by hypodermic injection (Nervous Diseases). He states that he has often given as much as 35 drops of Fowler's solution as an initial dose; and that when symptoms of poisoning have supervened when he has been giving it by the mouth, he has resorted to hypodermic injection, with the result that all gastric symptoms have ceased. Frühwald treated 22 cases of chorea also with 5 to 20 drop doses of Fowler's solution with good effect (Jahrb. f. Kinderheilkunde, 1887).

### MONOXIDE OF COPPER IN TAPE-WORM.

Dr. Schmidt, a Russian physician, has had considerable success in expelling tape-worm from himself and other patients by means of black oxide of copper (CuO). He used it as follows: Black oxide of copper, gr. 90; prepared chalk, gr. 30; clay, gr. 180; glycerine, ʒ ii ss. Make into 120 pills; sig. 8 to 12 pills daily. Two pills are taken four times daily for a week, and three or four times daily for another week, the patient abstaining meanwhile from all acid food or drink. At the end of two weeks a large dose of castor oil is taken to expel the worm. In the case of children the cupric oxide may be made up into lozenges—1½ grains in each lozenge, and two or three lozenges to be taken daily. Dr. Schmidt experienced some slight pain in the iliac region during the first two days of treatment. From the fourth day numerous segments of worm came away in the stools, and the castor oil expelled numerous fragments. There was no relapse.

